# Commentary: Is there a role for diabetes-specific nutrition formulas as meal replacements in type 2 diabetes?

**DOI:** 10.3389/fendo.2022.1094954

**Published:** 2023-01-18

**Authors:** Andrea J. Glenn, Simin Liu

**Affiliations:** ^1^ Department of Nutrition, Harvard T.H. Chan School of Public Health, Boston, MA, United States; ^2^ Department of Nutritional Sciences, University of Toronto, Toronto, ON, Canada; ^3^ Toronto 3D Knowledge Synthesis and Clinical Trials Unit, Clinical Nutrition and Risk Factor Modification Centre, St. Michael’s Hospital, Toronto, ON, Canada; ^4^ Centre for Global Cardiometabolic Health and Department of Epidemiology, Brown University School of Public Health, Providence, RI, United States; ^5^ Division of Endocrinology Department of Medicine, The Warren Alpert School of Medicine and Rhode Island Hospital, Providence, RI, United States; ^6^ Department of Medicine, and Division of Cardiothoracic Surgery, Department of Surgery, The Warren Alpert School of Medicine and Rhode Island Hospital, Providence, RI, United States; ^7^ Department of Epidemiology, Harvard T.H. Chan School of Public Health, Boston, MA, United States

**Keywords:** meal replacement, weight loss, diabetes prevention, diabetes remission, nutrition

## Introduction

Modest and sustained weight loss has been shown to reduce the need for glucose-lowering medications and improve glycemic control in overweight and obese individuals with type 2 diabetes (1–3). Still, these individuals face challenges in achieving and maintaining weight control due to a plethora of metabolic, psychological, and behavioral factors (4, 5). The use of liquid meal replacements within a structured dietary plan may offer a viable solution. Liquid meal replacements provide a mixture of carbohydrates, fat, and protein, along with added vitamins and minerals, in ready-to-drink form or powder formulas that require mixing. They are frequently used to replace one or two main meals each day, or in some cases, all meals (i.e., total diet replacement [TDR]). Current gaps in this area of research include identifying the type of formulations and complimentary aspects of a dietary program that yield long-term weight loss maintenance, reduce cardiometabolic risk, and increase the odds of diabetes remission in overweight and obese patients with type 2 diabetes. In this special issue in the *Frontiers of Endocrinology*, we comment on three key perspective articles that outline (1) the role for diabetes-specific nutrition formulas (DNSFs) as meal replacements in type 2 diabetes management (6), (2) lessons learned from the Diabetes Remission Clinical Trial (DiRECT) which demonstrated diabetes remission with the use of TDR followed by structured food reintroduction and long-term weight loss maintenance (7), and (3) use of meal replacements as a temporary option to induce weight loss followed by transition to a dietary pattern that aligns with the values, preferences and treatment goals of the patient (8). In the first article, Noronha and Mechanick highlight that DSNFs can be used to deliver essential macro- and micronutrients that align with the core nutrition principles from clinical practice guidelines for diabetes prevention and management (6). Compared to standard formulas, DSNFs have different macro- and micronutrient compositions to help manage dysglycemia, malnutrition and cardiometabolic risk factors. For example, they may contain lower glycemic index carbohydrates, added fiber, different fatty acid profiles and more protein. As outlined by the authors, there may be benefits to using DSNFs in patients with type 2 diabetes compared to standard formulas and standard test meals (i.e., cornflakes and milk/oatmeal) (6). Several acute randomized controlled trials (RCTs) showed that DNSFs significantly lowered postprandial glucose and insulin excursions compared to the standard control (9–13). Furthermore, the authors noted a recent pilot study that evaluated using DNSFs twice a day (breakfast and/or afternoon snack or before bed) compared to participants consuming their habitual diets (14). The DNSF breakfast/afternoon snack group showed improvements in glycemic control, including nocturnal glycemic variability, and resulted in other behaviors that may be valuable for cardiometabolic risk reduction such as experiencing less cravings for starchy foods.

Longer-term studies that include DNSFs as part of the intervention have additionally shown health benefits in patients with type 2 diabetes. For instance, a 6-month study implemented the transcultural diabetes nutrition algorithm (tDNA), which includes a structured low-calorie meal plan with motivational interviewing (MI) or conventional counseling, compared to conventional care (15). The study found that those in the tDNA MI and conventional counseling group decreased their HbA1c by 1.1% and 0.5%, respectively, and lost ~7kg and ~5 kg body weight, respectively, with no differences in the conventional care group. Moreover, in the Look AHEAD Trial, those assigned to an intensive lifestyle intervention group that incorporated various meal replacements including DNSFs, had greater improvements at 4 years in weight loss, HbA1c, blood pressure and high-density lipoprotein cholesterol (HDL-C) compared to the control group (16, 17). After 8 years, a clinically meaningful weight loss ≥ 5% was still observed in 50% of the patients in the lifestyle intervention group (18). These findings have been corroborated in several systematic reviews and meta-analyses where DNSFs improved glycemic control by reducing postprandial glycemia and HbA1c, without adverse effects on blood lipids, with some even showing improvements in triglycerides (TG) and HDL-C (19–21).

In the second article, Noronha et al. discuss how body fat accumulation is the dominant driver of type 2 diabetes, and therefore recommend weight loss as the best solution to reduce progression from prediabetes to type 2 diabetes or to achieve remission of type 2 diabetes (7). For remission to occur, weight loss of > 15 kg is generally needed. Bariatric surgery is an option to obtain this level of weight loss, however, this surgical procedure may not be appropriate for all patients (7). Another option that can be utilized is a low-calorie TDR. A trial that examined this type of intervention was the DiRECT trial, which investigated whether a weight management program delivered within primary care could achieve weight loss needed to sustain remission of type 2 diabetes (22, 23). Diabetes remission was defined as an HbA1c <6.5% without glucose-lowering medication for at least 3 months. During the TDR phase, patients consumed a micronutrient replete liquid formula diet (825 to 853 kcal/day) in place of their regular diet for 12 weeks, and discontinued their glucose-lowering, antihypertensive and diuretic medications. From weeks 13 to 19, patients slowly transitioned to a food-based weight management diet. Lastly, during weeks 19 to 104, patients followed a food-based diet based on the UK Eatwell guidelines with an individually tailored energy prescription with the option of consuming one liquid meal replacement per day. Medications were added back as needed. The DiRECT trial showed great weight loss success: during the TDR, participants had a mean weight loss of 14.5 kg, with an overall mean weight loss of 7.6kg at the 2-year follow-up. After 1 year, 46% in the intervention group achieved remission compared to only 4% in the control group. At 2 years, the remission rate remained at 36% (23). Importantly, the achievement of remission and those who had a decline in remission was directly related to the weight loss or gain among participants in the DiRECT trial (24). For example, participants who lost more than 15 kg had remission rates of 86% to 82% at 1 year and 2 years, respectively. Those who had a decline in remission from 1 year to 2 years had weight regain to within 10kg of baseline. In contrast, those who failed to achieve remission at both 1 year and 2 years lost less than 6 kg. Other significant developments in the intervention group included greater reductions in blood pressure and HbA1c, less use of antihypertensive and glucose-lowering medications, as well as better quality of life (22, 25). Along with these cardiometabolic improvements, a health economics analysis of the DiRECT trial showed significant healthcare cost savings (26). Another important development from DiRECT was that participants and healthcare providers found the TDR phase easier than expected, although the transition to regular food and weight maintenance phase was challenging and required more care (27, 28).

A nearly identical intervention study (DIADEM-I) also found significant amounts of diabetes remission in the intervention group (61% vs. 12% in the control group) (29), highlighting the findings were replicable in another population. Overall, the findings from DiRECT and other trials of surgical and non-surgical weight management interventions indicate that a weight loss of 10kg/%, ideally >15kg/15%, is needed for remission and that this should be accomplished as early as possible after diabetes diagnosis (7).

The third article focuses on the need to transition to a healthy dietary pattern from the temporary short-term use of meal replacements (8). The authors comment on a recent systematic review and meta-analysis using the Grading of Recommendations Assessment, Development and Evaluation (GRADE) approach to examine the role of liquid meal replacements on cardiometabolic risk factors in overweight and obese individuals with type 2 diabetes (30). This meta-analysis showed that there was moderate certainty of evidence for reductions in body weight, body mass index, body fat, fasting insulin and systolic blood pressure, low certainty evidence for reductions in waist circumference, HbA1c and fasting glucose, with no effect on blood lipids, in trials ranging from 1 month to 1 year. As liquid meal replacements are meant as a more temporary solution, transitioning to a healthy dietary pattern for long-term weight maintenance and cardiovascular health is crucial. The authors highlight two heart healthy dietary patterns as potential long-term solutions (8). The first dietary pattern discussed is the Mediterranean diet, characterized by high consumption of fruits, vegetables, legumes, nuts, whole grains and olive oil and moderate to low consumption of animal products and wine. The Mediterranean diet may be a beneficial diet for those with type 2 diabetes to transition to after weight loss as it has been shown to reduce cardiovascular events by ~30% in the The PREvencioín con DIeta MEDiterraínea (PREDIMED) trial (31), and a meta-analyses of trials and prospective cohort studies have shown cardiovascular benefits in individuals with diabetes (32). The other dietary approach recommended by the authors is the Portfolio diet, which is a plant-based cholesterol-lowering diet that is high in plant protein (particularly soy), viscous fiber, nuts, plant sterols, and healthy oils, while being low in saturated fat and dietary cholesterol. This specific dietary pattern may be beneficial as a recent meta-analysis found that the diet significantly lowered the primary lipid target for CVD prevention, low-density lipoprotein cholesterol (LDL-C), as well as the alternate lipid targets, non-HDL-C and apolipoprotein B, among other cardiometabolic risk factors (33). The Portfolio diet has also been associated with lower incidence of cardiovascular events and type 2 diabetes in a prospective cohort study (34, 35). As emphasized by the authors, healthcare providers will need to align dietary patterns with the values, preferences and treatment goals of the patients to achieve long-term adherence and health benefits (8).

Overall, liquid meal replacements replacing 1-2 meals per day or all meals (TDR) may have cardiometabolic advantages over traditional weight loss diets in overweight and obese adults with type 2 diabetes, with DNSFs potentially providing even greater benefits for glycemic control compared to standard formulas (6, 8). Liquid meal replacements may likewise be a useful strategy to induce significant weight loss for diabetes remission (7).

## Conclusions

These three perspective articles highlighted in this special issue in the *Frontiers of Endocrinology* underscore the use of liquid meal replacements as an effective dietary strategy to induce rapid weight loss followed by the need to transition to a healthy dietary pattern to further improve cardiometabolic outcomes and reduce cardiovascular risk over the long-term. Potential mechanisms likely include the calorie restriction achieved with liquid meal replacements, as well as macronutrient distribution as some meal replacements may be lower in carbohydrates compared to habitual diets. Limitations of these products may include taste preferences of patients, cost and adherence to the diet. Future research should determine the clinical efficacy and effectiveness for long-term use of liquid meal replacement, particularly DNSFs, in large and clinically relevant trials of diverse populations. Several trials are registered on Clinicaltrials.gov which are further investigating the role of meal replacements in diabetes remission, including examining plant-based meal replacements, online programs, rural populations, among others that may not be available on this site (36). In addition, further training of healthcare providers to use liquid meal replacements for weight loss in clinical practice will be needed. Achieving weight loss is challenging for free-living adults, with weight maintenance (and therefore continued diabetes remission) being even more difficult. Thus, behavioral interventions that help manage psychosocial and environmental stressors, and encourage physical activity and healthy diets are essential (7). Weight loss medications, such as GLP-1 agonists in particular, are also emerging as critically important strategies in any multi-component weight management program that integrates a phase of TDR for glycemic control with substantial benefits on weight loss and cardiometabolic health outcomes. GLP-1 agonists may offer a more palatable approach to weight loss than meal replacements, however, the cost of these medications may be a barrier for some patients as well as possible unwanted side effects that would not occur with using meal replacements. The shift to a food-based diet after TDR was noted to be challenging in the DiRECT trial (28), therefore effective strategies, with incorporation of MI and adaptation to different cultures [as included in the tDNA (15)], are crucial to ensure successful weight loss maintenance.

## Author contributions

All authors listed have made a substantial, direct, and intellectual contribution to the work and approved it for publication.
